# Complexity Thinking as a Tool to Understand the Didactics of Psychology

**DOI:** 10.3389/fpsyg.2020.542446

**Published:** 2020-09-24

**Authors:** László Harmat, Anna Herbert

**Affiliations:** Department of Psychology, Faculty of Health and Life Sciences, Linnaeus University, Växjö, Sweden

**Keywords:** complexity theory, complexity thinking, education, didactics of psychology, learning, psychology teaching

## Abstract

The need to establish a research field within psychology didactics at secondary level has recently been voiced by several researchers internationally. An analysis of a Swedish case coming out of secondary level education in psychology presented here provides an illustration that complexity thinking—derived from complexity theory—is uniquely placed to consider and indicate possible solutions to challenges, described by researchers as central to the foundation of a new field. Subject matter didactics is defined for the purpose of this paper as a combination of general didactics and subject matter content, and considering the international nature of research traditions coming out of psychology, the implications of the results presented here cannot be regarded as limited solely to national concerns. An online survey was sent to secondary schools in Sweden. Discussions and lectures along with teaching to the book—alternatively used as inspiration—emerged as central from the thematic analysis of the results, providing the first mapping of teaching practices secondary level psychology in Sweden. An analysis, founded on complexity thinking—combined with a model enabling a delimitation of the scope of study—focused on time use and the importance placed on self-knowledge, along with the transformation of theory into practice. The former pointed to a teacher-centered nested subsystem (e.g., asymmetric relations between teachers and students), whereas the latter pointed to student-centered nested subsystems coming out of embodied knowledge (e.g., students as node) where psychological perspectives are learnt through self-reflection, case studies, and everyday life experiences (turning theory to practice), implying a holistic approach. The analysis applied to the Swedish case provides an illustration of how complexity theory has the potential to address challenges at the micro and the macro levels to the establishment of a new research field in psychology didactics and to indicate possible solutions (drawing among other things upon teaching experiences coming out of the Swedish case study). Psychology’s high relevance to everyday life, multi-causality, perspective pluralism, dynamic systems character, and scientific character make complexity thinking a relevant approach in the consideration of challenges to the establishment of a research field in didactics of psychology.

## Introduction

Subject matter didactics has developed out of general didactics and covers a variety of traditions, some aiming at methodology and teacher practices and others coming out of empirical investigations of student learning. Subject matter didactics developed historically as both teaching practice and normative theory (Sandfuchs, 1990; Tenorth, 2006), optimizing discipline-related learning processes, remain as pivotal features to this day (Cramer and Schreiber, 2018). However, the term is seldom used within Anglo Saxon traditions of research (Hudson, 2007; Kansanen, 2009) as is the case with general didactics (Kansanen, 1995, 1999, 2002; Hopmann and Riquarts, 2000; Westbury, 2000; Hudson, 2007). The term didactics is often considered to have negative connotations in the Anglo Saxon world (Kansanen, 2009) and, in an attempt to avoid this, a new way of spelling didactics has been introduced, *didaktik* (Hudson, 2007; Kansanen, 2009), along with subject matter *didaktik* (Kansanen, 2009). Didactics will, however, be used for the purpose of this paper; furthermore, subject matter didactics and subject didactics will be taken to have the same meaning (and psychology didactics will be used alternatively with didactics of psychology). The core of subject matter didactics has been described as “how to combine subject matter or content with general didactics and arriving at an optimal way to teach and study” (Kansanen, 2009, p. 32), an approach which will be adopted for the purpose of this paper. General didactics then is an important part of subject matter didactics.

Uljens (1997, p. 49) suggested that “the aim of didactics is to understand and structure the overall educational situation with intentionality as one of the fundamental notions in contrast to methodik—the object of instructional method”—which Uljens goes on to describe as concerned with “activities … required in order to support an individual’s learning” (Uljens, 1997, p. 49). In research, general didactics is often referred to as the science of teaching and learning, the science or theory of teaching, the theory of the contents of formation, the theory of the steering and learning process, and finally the application of psychological teaching and learning theories (Gundem, 2000, 2011). General didactics then covers a great variety of different fields; the same is true of subject matter didactics. A plethora of different research traditions and approaches have emerged, varying both between countries, within countries, across subjects, and within subjects (Englund, 2007; Buchardt and Osbeck, 2017).

The need to establish a new field of research—didactics of psychology—has been voiced recently by several researchers internationally, along with a description of the challenges to the project (Spinath et al., 2018; Tulis, 2018). Tulis’ proposal will be presented at the outset of this article. The challenges described are related both to general didactics and to the generic aspects of the subject, pointing to the possibility of establishing a meta-level of psychology didactics (e.g., not only applicable to German secondary education). The international nature of research traditions coming out of various perspectives within psychology (taught at school) makes the combination of general didactics and subject content well placed to provide an approach not solely limited to national concerns, e.g., a meta-level of subject didactics. These challenges proposed will therefore be considered pertinent to the establishment of secondary level psychology didactics in Sweden. A mapping of Swedish secondary level psychology education will be carried out for the purpose of this article and is the first of its kind. There has, however, been a discussion in Scandinavia regarding the possibility of presenting different meta-levels of subject didactics, what these might be, and how they differ to general didactics.

Coming out of Sweden, for instance, Brante (2016) argues that Klafki’s model of general didactics presents a framework for delimiting levels—including any problems or perspectives of interest to subject matter didactics—rendering subject matter didactics superfluous. Brante (2016) presents a review of a whole range of papers in different subject matter didactics to support this claim. Kansanen (2009) suggests that there may be a high level of similarity between school subjects in regards to didactics, and it may therefore be more propitious in certain instances to group didactics coming out of these subjects into fields such as subject didactics of arts, subject didactics of natural science, and subject didactics of practical subjects, providing a meta-level of didactics common to each group. Kansanen (2009), however, also argues that the best means of defining subject matter didactics is through a combination of didactics and subject matter and in so doing claims that there is always a field of general didactics within each subject matter didactic. Sjöström (2018) criticizes Brante’s position and presents a model for a meta-perspective within subject didactics partially inspired by Klafki’s theory of general didactics.

A search of scholarly articles shows no published research papers on subject didactics of psychology other than through references to educational psychology in Scandinavia and in Sweden. One chapter describing changes to the national curriculum in Sweden has been presented by Blåvarg (2018). Both Gundem and Hopmann (2002) and Hudson (2007) argue that there are significant differences between didactics and curriculum studies; the same approach will be adopted here (e.g., these cannot be considered interchangeable). However, subject matter didactics can include curriculum analysis as important to the development of teacher practices and facilitation of the delimitation of the subject matter to be taught in the classroom. For these reasons, Blåvarg’s chapter will be considered.

Davis and Sumara (2006) have suggested that complexity thinking coming out of complexity theory is a powerful conceptual framework when applied to education. The aim of the investigation proposed here is to discover if complexity thinking can provide possible solutions to challenges, described as central to the foundation of a new field when applied to a case study coming out of Swedish secondary level. Teacher practices will be considered more specifically—coming out of Nilholm’s (2016) proposals for *gestaltande didaktik*—as pertinent to a meta-level of subject matter didactics. Teachers in Sweden arguably face challenges similar to those described by Tulis (2018) if these indeed are pertinent to a meta-level of subject didactics, and any insights coming out of the Swedish case study should also be applicable to the German situation in return. For the purposes of the investigation at hand, a model inspired by complexity theory and Klafki’s theory of general didactics (Klafki, 2000) combined with complexity thinking will be used to facilitate for the delimitation of the study (e.g., micro and macro levels) and to consider dynamic interactions between and within levels. Tulis’ (2018) proposal concerning German psychology education will be considered at the outset of this article; a presentation of Swedish psychology education will follow, after which complexity thinking will be discussed, followed by a presentation of the model developed for the delimitation of this study. A methods section describing the collection of material for this case study based on an online survey sent to psychology teachers at secondary level in Sweden will be presented, along with a results section coming out of a thematic analysis. Complexity thinking will be applied to the case study at hand in the discussion so as to facilitate the multilevel analysis and to deal with not only different levels but also perspective pluralism in psychology. The complexity of interactions between factors affecting the teaching and learning relationship, such as the dynamic interaction of various systems at societal, organizational, and individual level, and also environmental factors are considered, followed by a section presenting conclusions.

### Subject Didactics of Psychology

Psychology is characterized by perspective pluralism, involving a variety of fields with different research traditions and cultures. In countries such as Germany, researchers have expressed an interest in developing subject didactics within psychology. Tulis (2018) cites the German Society of Psychology as having pointed to the “necessity to establish subject didactics psychology”. Spinath et al. (2018) claim that “There is no sound subject didactics either for psychology at universities or for psychology lessons in schools in Germany” and recommend that “Psychology should establish its own subject didactics” (Spinath et al., 2018, p. 12).

Tulis’ (2018) proposal for how a field might be developed includes *paradigm-oriented psychology didactics*, *integrative psychology didactics*, and *action-oriented psychology didactics*. In paradigm-oriented psychology didactics, the thinking concerning subject psychology and the respective explanatory patterns of different paradigms are clearly and didactically reduced and compared (Sämmer, 1999; Glassman and Hadad, 2013). Nolting and Paulus (2016) suggested an approach for integrative psychology didactics, which considers multiplicity and complex interactions. Their basic idea is the classification of psychic phenomena into a heuristic frame of reference, which constantly directs the view “to the whole” focusing on, namely, the interplay of personal and environmental factors influencing human experience and behavior. The action-oriented approach in psychology didactics is introduced by Ruthemann (2007), with its focus on student activity in the classroom and the competent application of specialist knowledge and methods in practice. Action-oriented psychology didactics also aims to restructure inadequate mental models, information coming from the “knowledge of everyday psychology”. These three approaches—normally applied within the field of educational psychology (both secondary level and tertiary level)—are brought in to deal with, among other things, pupils’ faulty application of concepts used in psychology education.

The main characteristic of subject psychology is arguably *perspective pluralism.* Thus, psychological issues can best be described, explained, predicted, or influenced when viewed or treated from different perspectives (e.g., from a behavioral, cognitive, and systemic point of view). Perspective pluralism is put forward as one of the central challenges of psychology teaching by Tulis (2018). Tulis also points to another challenge facing psychology didactics—in so far that psychology is a “soft science”—there is no “right or wrong,” only a collection of different perspectives and opinions as compared to perspective pluralism within the natural sciences, which does not invite to personal speculation in regards to epistemology or knowledge. Based on the main characteristics of the subject, Tulis (2018) proposed four fundamental challenges for psychology didactics:

(1) How is it possible to teach psychology in such a way that pupils do not consolidate “everyday/lay psychology” or “sluggish knowledge”?
(2) How can human experience and behavior as well as the associated approaches of the different sub-disciplines and theoretical streams of psychology be conveyed without losing sight of the whole?
(3) How can scientific psychological insights (i.e., the understanding of a specific cause and effect within a specific context) and specialized methods be acquired in an action-oriented way so that they can also be used or implemented in extra-curricular situations?
(4) How can theories, approaches, and procedures of different paradigms lead to learning psychology by example, through a comparison and through integration into the existing knowledge structures?

In consideration of the first challenge—the revision of existing everyday psychological assumptions—Hughes et al. (2013) argue that psychological misconceptions are relatively resistant to change. Empirical evidence of discrepancies or disagreements are not always sufficient to revise the idea of everyday experience (Duit, 1995). Considering the second challenge—e.g., difficulties in dealing with the integration of sub-disciplines and theoretical streams of psychology—Nolting and Paulus (2016) argue for the classification of psychic phenomena into a heuristic frame of reference. According to this approach, not only different phenomena but also the basics and the applications of psychology can be integrated into a superordinate model and networked with each other (Nolting and Paulus, 2016). In relation to the third and the fourth challenges, Seiffge-Krenke (1981) outlines three steps for the initiation and the control of learning processes in psychology lessons (p. 312). These include consistent attachment to the naive-psychological assumptions of the pupil’s awareness and reflection of these everyday psychological theories and their limits through the didactic principle of alienation and cognitive conflict—and learning goal-related restructuring through the use of specific teaching methods to modify existing assumptions.

Psychology didactics emerges as a complex phenomenon according to Tulis’ description of challenges to the field. The specific characteristics of psychology includes high relevance to everyday life, multi-causality or dynamic system character, scientific character, and perspective pluralism (Nolting and Paulus, 2016). Comprehension of psychology goes through an evaluative epistemological perspective, namely, that psychological findings must be critically reflected, weighed against each other, and evaluated or justified in terms of context and situation (Birke et al., 2016). Thus, what is central to describing or understanding (psychology) education as a complex system is the identification of the components, their interactions, and what emerges from the complex system. To address the challenges put forward by Tulis and the recommendations made by Spinath et al. (2018), an analysis using complexity thinking as an approach is presented.

### Psychology Education at the Secondary Level in Sweden

Psychology became a separate curriculum subject in Sweden in 1965 after the national revision which took place in the 1960s (Blåvarg, 2018). Previous to this, the subject had been present in various curricula and various contexts (e.g., part of philosophy, anthropology, and the subject of religion). However, after an extensive revision of the curriculum in 1994 (Skolverket, 1994), the subject of psychology became mandatory only for some programs such as health education and social science in Swedish secondary schools (Blåvarg, 2018). At the same time, the syllabus was divided into different courses: “psychology A” and “psychology B.” “Psychology A” focused on the basic theories of psychology and their everyday application; “psychology B” contained applied perspectives—i.e., psychology applied to societal and psychiatric perspectives (Blåvarg, 2018). In 2011, there was a complete revision of the Swedish secondary school system (Skolverket, 2011b). The subject of psychology was also revised and became mandatory in social science, health education, and economics programs. Three new courses were proposed by Skolverket (2011a), “Psychology 1” covering the basics of psychology, and “Psychology 2a” and “Psychology 2b,” adding advanced and applied approaches building on one another. Seven out of 18 national programs offer psychology as a subject in Sweden (Skolverket, 2020). Pupils are instructed to read a minimum of 50 points where the subject is optional or obligatory; it is, however, possible to read 150 points. Points roughly correspond to lecture hours in terms of time spent by teachers working with pupils on the subject, but allocated time to any subject varies between schools and is the prerogative of the principal. In total, pupils take around 2,500 points to become eligible to apply for tertiary education (Skolverket, 2020). In the context of a program then, the subject of psychology is fairly small. However, core subjects are often no more than 100 points per course, and pupils read a wide variety of different courses. For the social science program, which is one of the largest and most popular programs in Sweden, psychology is mandatory in all specializations (Skolverket, 2020). Approximately 40% of all pupils graduating from upper secondary schools have grades from “Psychology 1” according to Statistics Sweden (SCB, 2018), coming out of programs where the subject is either mandatory or voluntary due to the various tracks.

Psychology courses include an introduction of the history of psychology, including the emergence of psychoanalysis and behaviorism, as these are requirements coming out of the national curriculum (Skolverket, 2011a, b). Pupils go on to read biological psychology, cognitive psychology, social psychology, and health in the first semester, followed by personality, developmental psychology, clinical psychology as well as studying the influence of media and culture on human behavior in the second semester (as part of “Psychology 2b” for those who opt to take a more advanced course). The third and the final course, “Psychology 2b,” enables pupils to apply their knowledge and specialize.

According to descriptions posted on the National Agency for Education’s homepage, the subject of psychology is intended to help pupils develop knowledge of factors influencing behavior, cognition, and emotion, both at an individual level and at a collective level (Skolverket, 2020). The course aims to increase self-knowledge through self-reflection, and teachers are directed to give pupils the opportunity to reflect on various psychological phenomena and perspectives. Psychology is also set to promote tolerance of difference by comparing people’s way of life, behaviors, and values and to develop critical approaches to different psychological perspectives and explanatory models. Theories are promoted as important in the description of the course’s specific content. It is emphasized that pupils must learn to use and evaluate different psychological theories and models and to merge different perspectives into a holistic view. Thereby, the assumptions expressed through the curricula seemingly promotes that different approaches taught in psychology can, to a degree, be considered complementary (e.g., do not present so fundamentally different views of human nature that these could not be merged into parts of a whole). The grading criteria are largely based on descriptive propositions, such as for the grade C in “Psychology 2b” where the student must describe and/or give a detailed account of different perspectives within personality psychology. Descriptive grading criteria give teachers a wide birth to define different kinds of examinations. Methods such as experiments and observations are only recommended in “Psychology 2b”. The subject then can, to a large extent, be covered by lectures and group assignments along with written examinations as directed by the curriculum (Skolverket, 2020).

The diversity of the set of relations described here implies that the didactic relation could not be organized universally or according to technical rules. One of the approaches which might help us to overcome the limits of the causal–effect explanation of some phenomenon in education research is complexity thinking (Davis and Sumara, 2006).

### Complexity Thinking and Education

Complexity thinking—derived from complexity theory—is a mindset used to frame a problematic situation, which can be identified as a complex system of interactions (Davis and Simmt, 2006; Davis and Sumara, 2006; Forsman, 2011). Complexity thinking has been applied in educational research, and it aims to describe and understand complex systems and their capacity to show order, patterns, and structure in educational activities (Davis and Sumara, 2006). Complexity thinking is not characterized by a particular research method but by a methodological perspective (i.e., a way of thinking) that employs a range of methods to study complex phenomena (Davis and Sumara, 2006). This approach is mostly used in research pertinent to higher education; however, the relevance of the approach may be useful in all educational settings to describe dissipative structures, the holistic and non-linear nature of learning processes, and the dynamical relations between agents taking part in the educational system (Morrison, 2002; Forsman, 2011; Forsman et al., 2014). Complexity thinking is a powerful alternative to reductionist approaches to educational science. Complexity thinking is an approach where deep similarities can be recognized among the structures and the dynamics of several disparate phenomena and can provide a useful tool to describe interpersonal dynamics involving teachers and their pupils in the classroom. Learning can, for instance, be interpreted in terms of recursive and elaborative interactive processes as opposed to cause–effect interpretations (Davis and Sumara, 2006). Doll (2008) discusses complexity theory and the culture of the curriculum pointing to how the aim of recursiveness often results in difference (the-yet-to-be-seen), especially if a curriculum is complex. However, according to some theorists such as Deleuze, repetition always leads to difference (Deleuze, 1968), so no classroom will produce the same behaviors and interactions in response to a set curriculum.

In general, complex systems are neither homogenous nor chaotic. They have structure embodied in the patterns of interactions between the components (Forsman et al., 2014). Some of these structures can be stable and long-lived (and are therefore easier to model), while others can be volatile and ephemeral. These structures are also intertwined in a complex way (Cilliers, 1998, p. 3). They can include neural networks, which are organic (Buzsáki, 2006, 2019), although there are certain advantages with structurally set networks. The term “complex” does not simply mean complicated but implies a non-linear relationship between components or agents and blurry boundaries between them (Buzsáki, 2006). Complex systems are open and information can be exchanged across boundaries, and small changes can cause large effects or no effects at all. For example, individuals who participate in education become active members of society, and some employ their influence on political decisions and culture, which, in turn, may effect changes in the educational system (Herbert, 2018). In this way, micro levels affect macro levels, and small scale affects large scale. Modern complexity science recognizes this as a *circular causality*, which may include multidirectional interactions across different levels of organizations (Nunez, 2016).

Stable structures can exist *a priori*, such as those of an organization with recursive activities as is seen in schools, where work is connected to the national curriculum (Carlgren and Kallos, 1997). The latter is recursive. These recursive systems can be described as nested, affecting subsystems, and can affect the structure on a lower level of nested systems such as those seen in a classroom. If a teacher, for instance, is working with a demanding curriculum (with many diverse goals and fields of study) and the time allocated for work is limited—there is a risk that the structure will be centralized and hierarchical, with the teacher at the helm of all activities (Herbert, 2018) to maximize production and the output of work (essays and examinations)—a teacher-centered network. If, however, a curriculum is less demanding and time is relatively liberal—decentralized networks with flat organizations allowing pupils to interact and direct activities can develop as a nested system—a student-centered network. These differences in systems and networks allow for different kinds of knowledge to develop. In this case, complexity theory emphasizes knowledge as emergent.

Phenomenon *emergence* means that new features arise through actions of smaller entities that do not possess these features in isolation. Emergence refers to novel or global properties that arise in complex systems from relatively simple interactions within a smaller system. *Knowledge* can also be described as embodied (Haraway, 1991), in which case pupils can be seen as nodes embodying knowledge. Embodied knowledge can also be seen to emerge from a network of neurons in interaction with the environment at both the micro and the macro levels. In so far that cognitive psychology and neuropsychology will be included in the understanding and learning about the brain, it can be argued that “knowledge” is emergent. Gazzaniga (2011) describes this form of “emergent knowledge” in his book, “Who Is in Charge: Free Will and the Science of the Brain.” Different sets of knowledge and thoughts “rise” to consciousness in competition with each other, and what any pupil/student remembers is the result of this competition, where certain sets of knowledge and memories gain precedence over others. This approach is also sympathetic with complexity thinking and the theory on which it is founded (complexity theory and network theory). Knowledge can also be *embodied* in things. Juelskjaer et al. (2013) describe, for instance, how the interaction between pupils, parents, and principals is affected by the couch placed in the principal’s office, requiring confessional practices of wrongdoing from absconding pupils called to meetings along with their parents. The microscope is, for instance, the result of knowledge developed over many generations, and pupils may learn about the history and the skills necessary to create it before using it. By handling a microscope and learning about a microscope, knowledge emerges and can be considered both in terms of behaviors (a laboratory examination) as well as cognitive capacity in finding new fields and new ways to use the microscope applied to the environment.

### The Complex System Model

**FIGURE 1:**
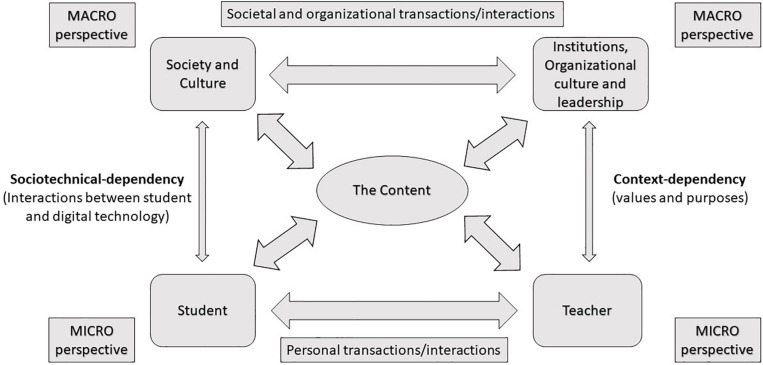
The complex system model.

While models are not ideal in the context of complexity science, they are often necessary (Cilliers, 1998). A combination of network theory and complex theory has been proposed by several researchers in the field (Cilliers, 1998; Morçöl and Wachhaus, 2009) and is considered of importance for public systems where both non-linear and linear processes are evident. In educational research, network theory has been used, for example, to characterize pupils’ interactions in small group discussions (Bruun, 2011) or to describe pupils’ retention from the school system (Forsman, 2011; Forsman et al., 2014). *Network theory* can be applied to explore, understand, and characterize structure connectivity in complex systems (Newman, 2018). Cilliers (1998) argues that the advantages of considering linearity in network models as constituting a part of a complex system is the possibility to describe both *stability* and *volatility* in structures. To be useful, these must have “some *a priori* constraints which will have to form a part of the interpretation of the results” (Cilliers, 1998, p. 8). Furthermore, a network must be “engineered in such a way that we know what it does…however, a model of the system would have had to exist beforehand in order to make the engineering possible” (Cilliers, 1998, p. 8). The linear model proposed here unfolds out of didactical traditions where the interrelation between student, teacher, and subject content playing out in the classroom is of central importance—as described in Klafki’s model of general didactics (Klafki, 2000; Hudson, 2007). Klakfi’s model is well established (Hopmann, 1997; Brante, 2016; Sjöström, 2018) and can be considered as *a priori*. The interactions between teachers, pupils, and the content (Hudson, 2003)—which can include the reader describing the topic or the technology facilitating access to information about the topic or subject—are depicted by a triangle with three points, with the sides describing different kinds of interactions which serve the purpose of this investigation where the classroom can be considered a complex system encompassing different kinds of nested systems (combinations between pupils, teachers, and technologies). Knowledge can be seen as emergent from the interactions (Gazzaniga, 2011). The nested subsystems can be of linear nature, and the interactions can correspond to the relations described in the didactical triangle. Complexity thinking is considered here to enable a better understanding of processes describing the interrelational dimensions of micro and macro levels, separately or otherwise, and Klafki’s interrelational model of general didactics has therefore been expanded with macro perspectives (Figure 1). The interactions between micro and macro perspectives are important in so far that the level at which psychology didactics should be developed is currently at issue. This model then aims to enable the consideration of a meta-level of general didactics, a meta-level general subject didactics as proposed by Tulis (2018), and micro levels of subject didactics—these perspectives are considered through an adjustment of the lens of delimitation enabled by complexity thinking in combination with the model, allowing for the possibility to scale up or down (from macro to micro and *vice versa*).

The model then has been developed for the purpose of describing and delimiting the field or scope of investigation—to a certain set of interactions—considered to be of importance, which have been descriptive and prescriptive, unlike complexity models, but can be used in conjunction with some linear perspectives as, for instance, those coming out of network theory. The structural dimension of the model corresponds to aspects of importance for an understanding of the structural and the logical relations between the agents. The temporal dimension corresponds to the time order of different types of activities—and interactions among agents at the macro and the micro levels—because most of the interrelations between the agents depend on “live interactions” within and between levels. Complexity thinking—coming out of complexity theory—presents a mindset used to problematize and consider solutions to the challenges described by Tulis. Primarily, complexity theory does, however, underscore “the importance of contingent factors of considering the specific conditions in a specific context at a specific time” (Larsen-Freeman and Cameron, 2008, p. 63) and, referring to historical examples, should therefore be seen as a means, whereby it is possible to understand a “humanitarian task of the present” (Klafki, 2000, p. 94), including education.

Complex systems are networked with other complex systems (Davis and Sumara, 2006). Moreover, components within a complex system can be considered to be complex systems themselves; thus, complex systems are nested. Nested systems have similar structure and dynamics but operate on different scales (time, size, and so forth) (Davis and Sumara, 2006). It is possible to consider society and organizations within education (i.e., schools) as two nested systems (at a macro level), which may have similar structures and dynamics. However, organizations in education are subsystems of the society, and these have been presented at the same level in order to describe the dynamic relationships with another, micro, level. The interactions on the micro and the macro levels may differ, considering their timescale (see above). Significant transformations at the macro level may take decades (e.g., changes in the curriculum), while significant transformations can take place over a few seconds at the micro level (e.g., discussions in the classroom). Hence, it is necessary to find relevant methods to measure the interactions on multiple levels in order to understand the interrelations between the macro and the micro levels from a dynamic perspective.

It is possible to describe the model proposed here of classroom interactions as a *self-organizing system* according to the sociological application of self-organization theory introduced by Luhmann (1995) (self-referential theory). For Luhmann, the elements of a social system are self-producing, i.e., “a communication produces further communications and hence a social system can reproduce itself as long as there is dynamic communication.” Self-organizing systems involving classroom interactions (four different discourses) have been described mathematically using the Scale Invariant theory (Herbert, 2018). The model above (Figure 1) can also be considered to be an assemblage. The assemblage theory provides a bottom-up framework for analyzing social complexity by emphasizing fluidity, exchangeability, and multiple functionalities (Deleuze and Guattary, 1987; De Landa, 2006). “Interpersonal networks may give rise to larger assemblages like the coalitions of communities that form the backbone of many social justice movements … social movements are a hybrid of interpersonal networks” (De Landa, 2006, p. 33) and “assemblages should be given the ontological status of individual entities: individual networks … individual cities and nation states” (De Landa, 2006, p. 40). However, emergence through self-organization has two directions: global-to-local and/or local-to-global determination [Haken’s system of synergetics referenced by Buzsáki (2006), p. 14]. In the system described here, we can find a dominance of the global-to-local determination. Global-order parameters (e.g., the curriculum and content, collective values, and purposes) may govern local interactions in the classroom, an interrelation described as *context dependent.* On other hand, factors which are *socio-technologically dependent* may also be evident, such as govern local interactions.

Complexity thinking can be applied together with the model described here (Figure 1) as a lens, which can be adjusted to delimit the field of study (from micro to macro and *vice versa*) to discuss the results of a mapping carried out for the purpose of this study in psychology didactics.

## Materials and Methods

### Contextual Information

Contextual information regarding the subject of psychology, the size of the topic—points given to the topic along with the number of pupils graduating with grades in the topic—was considered. An analysis of the national curriculum served as a preparation for the online survey.

### The Survey Content

Respondents were required to answer background questions related to age, gender, place of work, and educational background at the outset of the anonymous survey. These were followed by open-ended questions and multiple-choice questions (five-step Likert scale). The respondents were further requested to suggest ways in which psychology didactics at secondary level differs from other subjects. Questions designed to gauge respondents’ experiences of teaching were included in the first part of the survey. In the second part of the survey, the psychology teachers were queried in regards to common methods used in teaching practices as well as the technology used in the classroom (five-step Likert scale, ranging from 1, “not at all,” to 5, “very often”). Questions were presented separately for different levels of the subject (Psychology 1, Psychology 2a, and Psychology 2b.) The original survey was formulated in Swedish (see the English translation in Tables 1–6).

**TABLE 1:** What is psychology didactics for you?

Response category	Detailed description	Example
How to apply theories in practice	The teachers claimed that psychology didactic is a subject that helps a lot on how to apply theories in practice and how to teach their students to use these theories in their everyday life.	“The ability to turn theory into practice.” “That pupils see the impact of psychology on us in all aspects of our everyday lives. That pupils get to carry out practical exercises to reduce stress and performance anxiety and to manage groups.”
Elaborating pupils’ self-knowledge	The teachers described that subject didactic is helpful to them to find the proper method on how the pupils understand better one’s own and others’ emotions, thinking, and behavior.	“Getting pupils to better understand why they behave the way they do and why others do it.” “What influences us, how we think, behave, and learn, and how we become emotionally involved in different environments (e.g., learning environments and learning) and from the vantage point of different methods/perspectives.”
Explaining different psychological theories and concepts in a meaningful way	The teachers mentioned that subject didactic helps on how to explain different psychological theories and concepts sufficiently to their pupils.	“To approach different theories about man. To be able to work from the view that the various theories complement each other rather than provide complete truths.” “To test psychological theories and models together on real cases. By problematizing and discussing real cases, it becomes clear why there are several different theories and models.”
Elaborating the critical thinking of pupils	The teachers claimed that didactical methods are useful to problematize the knowledge in psychology in order to improve their pupils’ skills about critical thinking.	“Open the eyes of the pupils to be critical in their thinking.”
Short answers	The teachers gave just a simple answer and mentioned that they have never been thinking about this question.	“How to teach pupils in psychology.” “A pseudoscience like all didactics.” “Great question that I have not reflected on …”

**TABLE 2:** What is the difference between psychology didactics and the didactics in other subjects?

Response category	Detailed description	Example
More focus on theories and concepts	The teachers suggested that the subject contains more theories and concepts than other subjects deal with that.	“More focus on concepts and terms. Narrative, illuminating, *etc*.” “I think that, above all, Psychology 1 is very much tied to lecturing, but it does not have to be bad. There are many difficult theories and concepts that can be used as a teacher.”
Aim to increase pupils’ self-knowledge through self-reflections	The teachers claimed that, based on the curriculum, teaching psychology should give the opportunity to develop pupils’ ability to reflect on their own behavior and experiences.	“In the field of psychology, the purpose is to understand oneself and others and to reflect on one’s own behaviors and thoughts as well as their own feelings and thoughts, so didactically. I think it is important to have conversations, discussions, experiences, and not just have theoretical knowledge.”
More focus on connecting theory and practice	The teachers mentioned it is different in psychology teaching compared to other subject didactics, how to connect theoretical knowledge with practical knowledge.	“Psychology is very theoretical; my other subjects are more able to combine theory and practice.” “Maybe what is mentioned above with more focus on linking theory and practice. Which contributes to more experimental/laboratory-based means of teaching.”
Psychology teaching requires a special kind of methodology	The teachers claimed that special kinds of didactical methods are needed to problematize the knowledge in psychology.	“Trying to explain emotions, thoughts, and behaviors is different because it is so subjective and is close to people’s nature; it requires a certain kind of didactics/methodology.”
No difference (short answers)	The teachers claimed that there are no differences.	“Do not think that didactics differs very much from other theoretical humanistic subjects.”

**TABLE 3:** How the course books make an impact on your work with subject didactics?

Response category	Detailed description	Example
Inspirations for ideas	The teachers claimed that they get lots of inspiration on how to teach the subject in practice.	“They give me inspiration. Sometimes also a quick help for unplanned situations, such as completed study questions or reflecting/discussing/finding out assignments.” “They are important! They follow the central content of Psychology 1, 2a, and 2b. They address the central concepts in a good way and they provide inspiration for exercises and assignments in the course.”
Structure for teaching the subject	The teachers described that the books give the basic structure on how to present the material for their subjects.	“Good guidance.” “Use it mostly for reference in relation to the general course structure to create a basic structure.”
Support for methods and case studies	The books give support on how to implement different methods for teaching of the subject, and they can find useful case studies.	“It gives support to the areas we work with, discussion paper.” “Used as preparation for among other things, case histories.”
Support for teaching in general	The teachers just gave a simple answer about course books to support their teaching in general.	“The textbook supports teaching.” “They supplement with in-depth texts on different subject areas.”

**TABLE 4:** What is the biggest challenge for psychology teachers when the subject plan is to be transformed into didactic work in the classroom?

Response category	Detailed description	Example
Complain about time	The teachers complained that the time for their course is not enough to teach a complex subject with different concepts and their applications. They mentioned as the biggest challenge teaching psychology in Swedish secondary schools.	“The biggest challenge of all is that our courses are 50 points! With only 45 h, six different psychological perspectives must be covered (in Psychology 1).” “The fact that it is a 50-point course means that there is little time, which makes it difficult, for example, to give seminars and oral presentations when you also have large groups.” “To find time to cover the curriculum in a good way so that the pupils really learn.”
Applying psychological theories into practice	The teachers claimed that it is a challenge how to turn over psychological theories into practical knowledge in the classroom.	“Practical exercises. In-depth studies using only the textbook does not enable pupils to learn or understand. Getting more practical elements into education like experiments and labs.” “Finding relevant examples from everyday life.”
Explaining the connection between different theories	The teachers mentioned that it is a challenge how to make connections between the different subjects and theories in psychology.	“That the pupils’ interest and curiosity regarding the subject can be disturbed by the subject’s somewhat stilted division of perspectives (Psy1). At the same time, the subject plan is quite inexact/unclear regarding what theories to include.” “To go through as many different perspectives as possible so that pupils get a broad sense of the subject’s complexity.”
How to help the pupils to increase their self-knowledge	The teachers mentioned as a challenge in teaching of the subject how to help pupils increase their self-knowledge by learning the subject.	“That the pupils should see the connection between theories and reality; so that it will not be too difficult, they must get the real picture in order to relate the subject to themselves, for example.” “The problem is that it can be difficult to make space for the student’s own development throughout the course.”
Finding special methods to support the needs for individual differences and for the whole class	The teachers also mentioned that it is a challenge how to find the right method in the classroom to adjust the teaching material that follow the needs for the individuals and also for the whole class.	“To individually adapt everything to the pupils’ needs: teaching methods, examination methods, content, language, *etc*.”

**TABLE 5:** Which types of teaching methods do you use in the classroom?

	Psychology 1 (*n* = 61) %	Psychology 2a (*n* = 50) %	Psychology 2b (*n* = 21) %	Mean % (POMP)
Discussions	87	79	85	83
Lectures	79	84	71	78
Individual work	72	81	78	77
Written work	75	67	79	73
Practical exercises	78	74	64	72
Group work	62	67	63	64
Oral presentation	61	66	66	64
Seminaries	57	66	63	62
Workshop	42	36	54	44
Study visit	37	41	44	40
Laboratory session	34	34	45	37
Recorded lecture	32	37	42	37

*The sum of the rating scores on the five-point Likert scale in each method and the percentage of the maximum possible (POMP) scores, which expresses raw scores in terms of possible rating scores. n, number of respondents; %, percentage of maximum possible scores.*

**TABLE 6:** How often do you use the following methods for your teaching?

	Psychology 1 (*n* = 61) %	Psychology 2a (*n* = 50) %	Psychology 2b (*n* = 21) %	Mean % (POMP)
Case studies	79	80	63	74
Research articles	64	76	77	72
Digital tools	71	70	66	69
Films	67	67	60	64
Experiments	56	58	66	60
Observations	53	60	66	59
UR	60	60	57	59
Interviews	48	57	61	55
Science channels	53	53	54	53
Journals	51	60	46	52
Fictions	45	46	46	46
PBL	40	42	41	41
Role play	40	41	33	38
The flipped classroom	28	31	37	32
Logbooks	28	30	36	31
**Selection of the methods takes places**				
In a consultation with the pupils	52	76	70	66
In a consultation with other teachers	54	62	57	57

*The sum of the rating scores on the five-point Likert scale in each method and the percentage of the maximum possible (POMP) scores, which expresses raw scores in terms of possible rating scores. n, number of respondents; %, percentage of maximum possible scores; UR, Swedish Educational Broadcasting Company; PBL, problem-based learning.*

### Data Collection

A survey link was shared *via* email (Survey and Report, Linnaeus University). The public survey was sent out to 960 secondary schools in Sweden. While approximately 50% of these have social science programs, the subject is also taught on several other programs nationally in accordance with demand. This demand varies from year to year, warranting the choice of sending out the survey to a large group of secondary schools. Principals were charged with distributing the survey link to the psychology teachers responsible for teaching the subject. In addition, the survey link was shared with a special Facebook group which included 110 secondary school teachers in psychology in Sweden at the time of data collection. A reminder was sent out after 2 weeks of the first call. The participants’ answers were saved on a server maintained by Linnaeus University (Survey and Report). The written texts from the open-ended questions served as the basis for a thematic analysis of the survey content (Braun and Clarke, 2008).

### Data Analyses

A thematic analysis was applied to open-ended questions (Leininger, 1985; Aronson, 1994; Braun and Clarke, 2008). The application of thematic analysis demands that a sample population can be considered to be part of a community with shared concepts (Braun and Clarke, 2008), e.g., concepts used by the participants are understood and used in roughly the same way. The participants in the study are teachers with similar educational backgrounds working in school organizations and are therefore considered to fill the requirements for being part of a community with shared concepts and with shared meaning (roughly similar) given to these concepts. The responses to the open-ended questions of the survey were analyzed in order to identify categories or themes emerging from the material. A list of basic categories coming out of the text of each open-ended question in the survey (e.g., the responses to questions) was collated by one researcher. The list of basic categories from each open-ended question was then given to another researcher, who repeatedly compared the original list with text from the survey. These lists were repeatedly compared with the answers from the participants for these questions, until the two researchers agreed regarding the basic categories (Tables 1–4). In addition, the raw score for each participant’s answers on the five-point Likert scale was calculated. The raw scores from the five-point Likert scales were standardized in each question into the percentage of the maximum possible (POMP) scores which express the raw scores in terms of possible rating scores (Fischer and Milfont, 2010) (Tables 5, 6). The statements collected to open-ended questions are considered to be speech acts, where meaning can be inferred without considering the characteristics of the subject who utters the statement. The results coming out of this analysis act as a mapping or case study describing how psychology is taught and how subject matter didactics is conceptualized by 61 teachers in the field at the secondary level. Complexity thinking founded on the model prepared specifically to enable a delimitation of this study is then applied to the case created from the survey.

### Participants

Sixty-one psychology teachers (*M* = 44.5 ± 9.2 years old, 40 females, 20 males, one other) from schools in Sweden, who were responsible for teaching the subject at the secondary level, took part in the survey. The respondents had reliable educational background—teacher training at a tertiary level with a psychology major or a major in a related subject—along with extensive teaching experience in the subject (*M* = 11.6 ± 7.7 years of teaching expertise). There is no collated documentation as to how many teachers work with psychology at the secondary level, but given the size of the subject and the distribution of the social science program nationally, there are good grounds for treating this sample as representative (keeping in mind the limitations of the study).

## Results

### Open Questions About Psychology Didactics and Teaching of the Subject

Following background questions including age, gender, place of work, and educational background, questions regarding experience of education practices (see Tables 1–4) were asked. Coming out of the first question “What is psychology didactics for you?”, four response categories were found along with an additional category of “short answers” (e.g., named short answers). The first four categories can be interpreted as representing aims described in the curriculum such as the elaboration of pupils’ self-knowledge and critical thinking, the explanation of different theories, *etc*. Following on—from the question asking the respondents about the perceived difference between psychology didactics and other subject matter didactics—four categories emerged. These themes referred to the transformation of the curriculum into practice—i.e., a focus on theories and concepts, the aim to increase a student’s self-knowledge through self-reflection, a focus on connecting theory and practice—different approaches seemingly pointing to the necessity of developing teaching methods which differ from those of other subjects taught at the secondary level. The last category was defined as short answers (e.g., no difference). A third question investigated the impact of course literature (the reader) on teaching practices and work. The response indicates that teachers mainly use course books as a support to prepare for lessons—i.e., as an inspiration, a structure for teaching the subject, a support regarding methods and to provide case studies—as well as a more general support for education praxis in the classroom. A fourth question required the respondents to cite/describe challenges in regards to teaching practices. “What is the biggest challenge for psychology teachers when the syllabus is to be transformed into didactic work in the classroom?” One response category—emerging from the material—related to problems regarding time allocation. Teachers were challenged by having to teach complex material in the time allocated to the subject in the schedule, e.g., 45 lecture hours (Psychology 1). Other response categories were similar to those coming out of the second question (i.e., applying psychological theories into practice, explaining the connection between different theories, helping the pupils to increase their self-knowledge).

### Questions About Methods

In the third part of the survey, an inquiry was made into common methods used in the classroom. The sums of the rating scores on the five-point Likert scales in each method were turned into the percentage of the POMP scores, which express raw scores in terms of possible rating scores (Fischer and Milfont, 2010) (Tables 5, 6). The respondents were asked about teaching practices. Discussions, lectures, and individual work were listed as common classroom methods, and less frequent methods included study visit, laboratory session, and recorded lecture (Table 5). We also asked teachers how often they use other types of teaching methods. We found that case studies, research articles, and digital tools are commonly used, and role plays, the flipped classroom, and logbooks are used quite rarely for the purpose of teaching psychology. In addition, the selection of methods more often takes place after a consultation with pupils and not so often after a consultation with colleagues (Table 6).

## Discussion

Complexity thinking will be applied here as a means of enabling a dynamic approach for a better understanding of the relation between the micro and the macro levels. The combination of complexity thinking and the model developed specifically for this investigation (Figure 1) enables a delimitation of the field and scope of the investigation at hand. Further this combination facilitates for a discussion of the results of the mapping coming out of the thematic analysis which constitutes the case study. The first level analysed (micro level) involves a linear model which relies heavily on Klafki’s theory of general didactics and is considered to describe nested systems which are a priori.

### Micro Level

From the first mapping carried out of the results, it emerges that most combinations of methodologies favored by Swedish teachers are similar to combinations used in other subjects (e.g., lectures and discussion) and, in some instances, to certain groups of subjects such as natural sciences—experiments receive a mean score of 65%, observations receive 60%, and practical exercises. This gives some support to Kansanen’s consideration of creating larger groupings of subjects (Kansanen, 2009). Teachers describe practices faithfully mirroring the intention of the curriculum, informed by the values expressed there. In accordance with the curriculum, pupils are encouraged to learn theories and concepts as well as achieve self-knowledge through self-reflection, and to these ends, discussions and lectures are primarily used (Table 5). Teachers prefer to engage with pupils when transforming the curriculum into practice through the selection of methods rather than consulting colleagues (Table 6).

A dialogic approach related to the student–teacher axis of the model is implied here. The dialogic approach refers to an interaction between student and teachers, while the emphasis placed on self-knowledge through self-reflection points to a student-centered nested network where an adjustment of the focus enables a consideration of intra-action. The development and the integration of these two practices (self-knowledge and self-care) in educational context originated in Ancient Greece (Foucault, 1995). Self-knowledge is often described as the aim of maieutic (dialogic method) but is also related to the understanding of how the three parts of the soul (read here as psyche) could be balanced. This intra-action between the animalistic, the idealistic, and the realistic/rational parts is described by Plato in the Phaedrus (Plato, 1997, p. 506) and the Republic (Plato, 1997, p. 971), a model which has arguably inspired Freud’s topographical model (Freud, 1923), where the latter described as a chariot driver having to control two unruly horses can be conceptualized as a network (an “intra-action” coming out of a subject-specific approach when considering Freud’s modern theories). In Klafki’s model, self-care is integrated into formation or *bildung*. The subject of *bildung*, however, points only to conscious processes (e.g., precludes a consideration of intra-action between neurons, for instance, as enabled by the combination of network theory and complexity theory used for this analysis).

In regards to temporal factors, time is an issue according to teachers in the case study, especially where the transformation of theory into practice related to abstract models within psychology is concerned. An emphasis on lectures, as evident in this study, implies a more traditional uni-directive approach with asymmetric relations between students and teachers—(student–teacher axis of the model), which is common when logistical problems arise due to limited resources (Herbert, 2018). Fuite (2005) has hypothesized that the tendency of educators to perceive time as a limited resource may be one of the reasons for the emergence of centralized nested systems in the modern classroom where knowledge goes through the teacher (teacher-centered network).

The book is the primary technology through which both pupils and teachers achieve their goals and is depicted in the teacher–content axis. The book is mentioned as both enabling a foundation for teaching, an inspiration for teaching, and a means of teaching and is therefore yet another nested network of interactions describing classroom praxis, where the book becomes the central node. The centrality of the book, as indicated by the responses coming out of the survey, is likely to be a feature of many different subjects and cannot therefore be considered to characterize a meta-level of subject didactics.

Some of the books listed by Swedish teachers in this study, however, include case studies, and teachers often use these in classroom activities. Digital tools, practical exercises, and films are also used, but to a lesser extent than case studies (Table 6). The book in combination with methodologies promoting self-knowledge, including case studies, is one of the activities carried out to reach goals in the curriculum according to teacher statements, and case studies (as described in the psychology books listed by teachers) are particular to traditions coming out of psychology. Based on results coming out of the qualitative survey (Table 3) then, case studies are useful to elaborate a student’s self-knowledge and transform theory into practical knowledge. Case studies coming out of the reader along with the aim of self-knowledge (described in the national curriculum) and coupled with self-reflection are combinations which could arguably present a potential for a subject-specific approach (interaction of several nodes describing a new nested system).

There is a tension described here between the two nested networks (i.e., student-centered networks and teacher-centered networks) which can emerge from interactions at the micro level. The findings of this analysis mainly describe practices related to general didactics. Issues involving time, for instance, are not specific to psychology education (Fuite, 2005). The same is true regarding challenges involved in the transformation of theory to practice, which is arguably common to all theory-laden subjects including philosophy, mathematics, and physics. However, the emphasis on a combination of self-knowledge and self-reflection put forward by teachers in the mapping carried out for the purposes of this investigation describes practices within psychology education facilitated by case studies coming out of the book, and these could potentially strengthen arguments for a meta-level of subject didactics. We will move on to consider what complexity thinking enables in terms of a deeper understanding of interaction of networks described here as well as an interaction between the micro and the macro levels.

### Interpretation of Interaction Between the Micro and the Macro Levels in Education As Two Nested Systems

It is assumed here (e.g., implicit to the model) that interrelations at the micro level are not separate from interrelations at the macro level. There is a reciprocal relationship between the parts and the whole, and there are multiple causes for the changes in the system. Some previous research has focused on interrelations at a micro level by describing interactions between teachers and pupils (e.g., Bruun, 2011; van Geert, 2014). In Sweden, curriculum studies have been included in didactics as part of an attempt to understand the development of subject didactics. Swedish curriculum research has also been related to the consideration of macro perspectives, where changes in the Swedish school system have been discussed as well as political, social, and economical changes covering the late 1960s to mid-1990s (Carlgren and Kallos, 1997). These authors analyzed which societal groups dominate and influence the development of the curriculum during a specific historical period as well as why a certain kind of curriculum becomes possible in relation to a specific set of described circumstances. The analysis of how a curriculum expresses educational policy over time and what changes/interactions take place over an extended time frame can be applied to research at a micro level, e.g., interactions between teachers and pupils. Changes to the curriculum have multiple causes, which cannot be understood as linear and are better described as an accumulation of different events, a pile (Davis and Sumara, 2006, p. 89). Complexity science is a useful approach for the analysis of interactions between micro and macro level coming out of such changes. The model proposed here (Figure 1) allows for an understanding of temporal and structural dimensions of didactics. At a micro level, pedagogical inter-action is a shared activity between two subjects (teacher and student) focused on a specific content aiming at reaching goals commonly agreed upon (Uljens, 1997, p. 54). However, interaction at the micro level needs to be interpreted in a societal, cultural, and historical context, e.g., considering interactions at a collective level.

One example, following the transformation of the curriculum of the subject of psychology in Sweden, will be described here. Blåvarg (2018) describes changes within the subject in regards to how the pupils were involved (or not) in the teaching of the subject, as seen over the past half century. Based on the first curriculum in 1964, when psychology became an independent subject in Swedish secondary schools, pupils’ own life experience was taken as a starting point to learn the subject. After a change in the curricula (Skolverket, 1994, 2011a, b), focus was placed on abstract content in the subject matter (e.g., psychological). While a student’s self-knowledge was retained as an important goal in the curriculum, student experiences were no longer treated as the main point of departure, which might possibly explain the teachers’ perceived lack of time for teaching the subject as expressed in the responses to the online survey. The pressure to cover a broad curriculum in a limited time frame and the reduction of classroom autonomy may result in a system where all information is made to pass through a central hub (the teacher). It may also offer an explanation as to why the teachers in this study often use lectures or discussions (led by the teacher) as a method, instead of laboratory sessions or recorded lectures (Table 5).

During the late 1960s, most of the school system in Sweden was centralized by the state, but toward the end of the 1970s, a shift toward decentralization was carried out. Governance of schools was given to the municipalities or to private organizations. This transition from national to local governance of the schools in the 1980s was also in line with new ideological approaches such as neo-liberalism and neo-conservatism (Carlgren and Kallos, 1997). An emphasis on the individual’s self-reflection and self-knowledge as opposed to understanding in terms of a collective experience and society may be understood in this context. In addition, Sweden became a member of the European Union in 1995, resulting in educational systems becoming increasingly globalized. All of these complex issues may have impacted the changes in the curriculum at a national level, affecting the subject of psychology as, for instance, can be seen in aims to promote tolerance, awareness of difference, and understanding of social change (Psychology b). Further societal changes impact working conditions, digitalization, and fluidity; new markets result in new demands on pupils—coming out of educational institutions looking for work—creating shifts in what might be called the concept of the model citizen. The national curriculum (Skolverket, 2011b) of psychology points to the education of a citizen accepting of change, adjusted to a multicultural society, and prepared to take part in democratic processes. However, the latter aim is also part of general didactics as described by Klafki (Herbert, 2018).

### Emergence of Knowledge and Self-Knowledge

The classroom has been delimited as a complex system/network at the micro level and nested with in the macro level (Figure 1) in accordance with the model developed specifically for the purposes of this study. It is posited here that the macro level has top-down control on the micro level through the curriculum (context dependency) and through the implementation of technological and digital development (socio-technological dependency). Teachers must follow the updates of the actual curriculum, and all changes in the subject curriculum are set to act as triggers, with repercussions in the classroom affecting interaction and the emergence of knowledge (Gazzaniga, 2011).

Knowledge is considered to be *embodied*—in the model proposed here—in so far that the “self” is the central hub, which carries knowledge based on previous interactions with other nodes from which knowledge becomes an emergent property. Knowledge can be seen as an emergent property of complex interaction in bundles of neurons, expressed through language, for instance (Beckner et al., 2009), affected by the complexity of interactions which develop between teachers and pupils during lectures and discussions (Doll, 2008), which in turn can be affected by changes to the curriculum which emerge from the societal level involving complex interaction within politics.

Self-knowledge refers to how individuals understand their own character, feelings, motives, and desires. Self-knowledge requires ongoing self-awareness and self-consciousness through self-reflection. Interactions between micro and macro perspectives can be seen in so far that the pupil, imbued with knowledge of the self, achieves this through different praxis involving lectures, discussions, and self-reflection—implying not only a student-centered network coming out of the interaction but also a consideration of intra-action, adjusting the lens of complexity thinking to focus on internal nodes—the self and the I in the case of Mead’s models (Mead, 1934), the id, ego, and super ego coming out of Freud’s theory (Freud, 1923)—alternatively, neurological networks with no central hub can be considered, where knowledge is seen to be emergent as is described by Gazzaniga (2011). The pupil is directed to integrate different fields of psychology into a holistic approach through their own experiences (turning theory into practice), enabling an understanding relating individual experiences to group experiences and further on to experience at the societal level. Furthermore, the pupil may integrate a variety of separate fields of psychology with their own traditions, expertise, and research methodologies into a whole. Self-knowledge through self-reflection implies that a pupil-centered network can co-exist, nested within a teacher-centralized network in so far that self-reflection can be taught as self-care or care of self (embodied knowledge). This combination originates from educational practices in Ancient Greece (Foucault, 1995; Herbert, 2018). Self-knowledge (enabled through self-reflection coming out of contemplation, meditation, and prayer) aimed, among other things, at facilitating a balance between the three levels of the psyche or soul and could therefore be considered a pedagogical approach. However, this particular means of achieving self-knowledge was not adopted by any pedagogic philosophers or applied to pedagogy; instead it was adapted to Freud’s topographical model and can thereby be considered as subject specific (Freud, 1923).

The model presented here allows for complexity thinking to consider individual nodes (pupils) and embodied knowledge, describing this delimitation. Practices developed in the classroom to promote self-reflection are not limited by the classroom. Complexity thinking combined with the model developed for the purpose of this study merges both linear and non-linear, dissipative structures, allowing the analysis here to account for how self-reflection as a practice can carry over to other spaces. Self-knowledge then, developed through self-reflection, can be considered to translate into intra-action where pupils’ embodied knowledge is central.

The complexity of the aims of psychology education indicates that not only is psychology charged with “producing” future citizens with the espoused correct values, tolerance, and understanding of fellow man [typical of general didactics described by Klafki (2000)] but also the pupil must also be capable of holistic thinking by integrating the whole of psychology with all of its major fields, and this is achieved through, among other things, self-knowledge facilitated by case studies coming out of traditions in psychology [which may offer insights and possible solutions to challenges 2 and 4 as proposed by Tulis (2018)]. Self-knowledge has been described as belonging to a meta-level subject didactics (Herbert, 2018). As suggested by Birke et al. (2016), Swedish pupils are encouraged to think critically as a means to facilitate for comprehension of psychology, whereby psychological perspectives can be compared and findings within these perspectives can be critically reflected (weighed against each other and evaluated in terms of context and situation). Furthermore, a critical approach in combination with self-knowledge can be a means of challenging and changing the establishment of popular and faulty use of psychology concepts and theories. However, Swedish teachers did not raise this particular challenge as an issue.

### The Generalizability of the Study

A majority of Tulis’ (2018) challenges seem to be applicable to other subjects—in so far that the challenges are general (transforming theory into practice, for instance, along with the focus on critical thinking), the didactical frame from which they originate may also be considered general in line with Brante’s claims (Brante, 2016). However, subject didactics can be seen as a combination of subject matter considerations and general didactics (Kansanen, 2009). Subject matter content specific to psychology is evident in both Tulis’ challenges and those described by Swedish teachers as, for instance, the consideration of behavior and human experience in challenge 2 and the importance of behavior, emotions, and cognition (thinking) mentioned by Swedish teachers in regards to what differentiates psychology didactics from other subjects. Self-knowledge through the practice of self-reflection facilitated by case studies offers a potential solution to several challenges and the potential for a subject-specific approach. Complexity thinking based on complex network theory suggests that phenomena must be looked at holistically, and this enables multiple causalities, multiple perspectives, and multiple effects to be charted. We argue that complexity thinking combined with the model (proposed here), which aims to delimit the field of investigation—enables an understanding of subject didactics of psychology. Complexity thinking coming out of the model proposed here then can be applied to general didactics (i.e., it can be relevant for any pedagogical situation) and subject didactics (at different levels) that deal with teaching different subjects in organizations/schools due to the possibility to adjust the focus and the field of delimitation.

Complexity thinking does not aim to fix a focus on either subject matter didactics or general didactics, but in this study, we have aimed to discuss the challenges, described by teachers, to the development of subject matter didactics at the secondary level in Swedish education and use this to consider possible solutions to challenges voiced by researchers concerned with establishing a research field and *vice versa*. The analysis carried out here does lend support for the possibility of establishing a meta-level of subject didactics as proposed in support of Sjöströms’ proposal (2018) but does not definitely preclude Brante’s proposal (2016), complexity thinking points to a variety of possible delimitations of levels. Swedish teachers experience similar challenges as those described in Tulis’ proposal. Furthermore, the way in which these challenges were met by Swedish teachers does bring insights of importance to the proposal set forward by Tulis (2018), pointing to the generalizability of the results of the investigation carried out here.

Complexity thinking combined with the model presented here can be applied to understand and discuss questions and challenges to the establishment of subject didactics at the micro levels as well as meta-levels along with challenges faced at different levels of education, including tertiary levels. However, one must assume that there is still no definite and widely accepted answer to the question of how subject matter didactics relates to neighboring disciplines like educational sciences and pedagogy; the sheer number and the diversity of models used to describe subject matter didactics make this point.

### Suggestions for Future Research

Suggestions regarding possible fields for future research coming out of the model used for the analysis of the case of psychology teaching at the secondary level in Swedish schools will be presented here. It is proposed that complexity thinking is well placed to address several dimensions of psychology didactics which have emerged as important in the investigation carried out here, along with the challenges described by Tulis. Research can be carried out at the micro level (measuring classroom interactions, effectiveness of different teaching methods) and at the macro level (curriculum studies) separately. An emphasis is placed on the importance of taking a holistic approach (or understanding “the whole”) in this article. Society is currently complex. It is a flexible fluid society (Bauman, 2003), and it is important for individuals to be able to adapt to quick changes and to learn until the end of life.

In this study, the necessity of establishing a research field within secondary level psychology didactics has been pointed to. However, the role of research is seen to relate to both informing development and underpinning professional practice in relation to two main aspects: firstly, educational work in general and, secondly, in specific subject matter didactics (Hudson, 2007). What characterizes psychology didactics, setting it apart from other subject didactics, according to the investigation presented here is, among other things, the focus on behaviors, emotions, cognition, and an emphasis on self-knowledge as coming out of the case study of Swedish teacher practices, the translation of curriculum goals involving the demand that pupils should learn, and transform theories within psychology into practice through self-reflections in order to increase their self-knowledge, which in turn requires special teaching methods (in order to reach the goals of the curriculum). It is suggested here that future research focus on the means of increasing pupils self-knowledge and consider what kind of environment, methods, equipment, and support determines the emergence of self-knowledge in the classroom?

Learning about different theories and approaches in psychology may have an important role in improving pupils’ critical thinking. The question also arises regarding student-centeredness (decentralized control) as opposed to teacher-centeredness (centralized control) in the classroom (Davis and Simmt, 2006). If the classroom works as centralized hub (teacher-centered network), pupils may not have enough autonomy to think freely or share their experiences and different opinions with each other (it may even hamper critical thinking). Questions could be asked, such as: How is it possible to give more autonomy to pupils in secondary schools to learn the subject in a more decentralized way in the classroom but to avoid consolidating their “lay experiences”? How can teachers introduce complex theories in psychology and their relations to each other? What role might creativity have in a consolidation of this approach? How can concepts and approaches be interpreted by the teachers as related/or not related to the goals of the curriculum?

In response to Tulis’ claims regarding challenges to the foundation of a new research field within secondary level psychology didactics, we suggest that future research should also take into consideration how the basics and the applications of psychology can be integrated into a superordinate model and networked with each other. This could be helpful not only for the pupils but also for their teachers. According to Davis and Simmt (2006), research must also consider teachers’ knowledge about different concepts in psychology and their knowledge about how these concepts are developed and come to interact. In line with this aim, we suggest that future research also focuses on the complexity of the teachers’ knowledge and their pedagogical content knowledge (PCK) (Shulman, 1986, 1987; Nilsson, 2013, 2014; Nilsson and Karlsson, 2019). PCK suggests that teachers need pedagogical content knowledge, a special kind of knowledge teachers develop about how to teach a particular content to particular pupils. Teachers need to find appropriate methods and facilitate interactions in the classroom in order to teach theories and deepen their pupils’ self-knowledge through introspection, etc. PCK can supply or result in an important understanding of how teachers present and interpret these concepts to their pupils and how they relate that to their pupils’ learning.

Finally, further research at the macro level should focus more on complex changes in society (educational policy) and how these transformations may determine changes in the curriculum (Carlgren and Kallos, 1997). However, research at the macro level should not be thought of as separate from that at the micro level. We also need to understand emergence at the micro level, i.e., in the classroom (subject didactics) in regard to changes at the macro level. As we suggested, the classroom works as a complex system/network itself and nested with the macro level. Macro level has top-down control on micro level through the curriculum (context dependency) and through implementing technological and digital development (socio-technological dependency). However, multilevel descriptions are required for understanding because the macro level influences on the micro level and *vice versa*, demonstrating the process of circular causality (Nunez, 2016, p. 72). In addition, the connection between socio-technological development and how digital pedagogy should be implemented into psychology teaching in the secondary schools and higher education levels are relevant issues (Goldman-Segall and Maxwell, 2003).

## Conclusion

We interpreted the results coming out of a survey sent to psychology teachers in Swedish secondary schools (the first mapping of teacher practices within a secondary school of its kind) and the emergence of challenges in teaching the subject using thematic analysis.

Swedish teachers experience similar challenges as those described in Tulis’ (2018) proposal. Furthermore, the way in which these challenges were met by Swedish teachers does bring insights of importance to the proposal set forward by Tulis (2018). Knowledge development is described here as emergent from interactions between teachers and pupils as well as important artifacts such as books and technology, even words (Dahlbom et al., 2002). Further knowledge is discussed as embodied. The Swedish curriculum is considered here along with a brief presentation of how Swedish teachers currently “translate” the curriculum into practice. Complexity thinking, combined with a model allowing for a delimitation of the field of study, was applied to a case study coming out of secondary level psychology education in Sweden, focusing on the importance of self-knowledge (subject specific) along with the transformation of theory to practice (general didactics). The former points to a teacher-centered nested subsystem with asymmetric relations with pupils, and the latter points to a student-centered nested subsystem coming out of embodied knowledge (e.g., pupils as nodes) where psychological perspectives are learnt through self-reflection, case studies, and everyday life experiences (turning theory into practice), thereby implying a holistic approach.

If a field specific to psychology didactics—a meta-level of subject didactics as suggested by Sjöström (2018)—the emphasis on behaviors (possibly also emotions and cognitions) as well as self-knowledge could be a possible way forward. The analysis applied to the case study at hand enabled a consideration of issues regarding both micro and macro levels of didactics (concerns regarding both subject content and general didactics were evident) and illustrated how complexity theory has the potential to address the challenges described by international researchers and indicate possible solutions.

In this study, challenges to the foundation of a new research field within secondary level psychology didactics have been considered—the results presented here point to possible solutions—providing some support for the proposal, along with possible ways forward, in regards to the development of the field, not solely limited to national concerns. There is a need for broad research in psychology didactics, where complex issues need to be taken into consideration at the micro and the macro levels in didactics presented in our model (Figure 1).

## Data Availability Statement

The datasets generated for this study are available on request to the corresponding author.

## Ethics Statement

Ethical review and approval was not required for the study on human participants in accordance with the local legislation and institutional requirements. The patients/participants provided their written informed consent to participate in this study.

## Author Contributions

LH and AH contributed to the conceptualization of this study, formal analysis of data, investigation/experiment design, methodology, visualization of tables and figure, and writing of the manuscript. LH took charge of data curation and project administration. Both authors contributed to the article and approved the submitted version.

## Conflict of Interest

The authors declare that the research was conducted in the absence of any commercial or financial relationships that could be construed as a potential conflict of interest.
